# Yeast Sex: Surprisingly High Rates of Outcrossing between Asci

**DOI:** 10.1371/journal.pone.0010461

**Published:** 2010-05-05

**Authors:** Helen A. Murphy, Clifford W. Zeyl

**Affiliations:** Wake Forest University, Winston-Salem, North Carolina, United States of America; Texas A&M University, United States of America

## Abstract

**Background:**

*Saccharomyces* yeasts are an important model system in many areas of biological research. Very little is known about their ecology and evolution in the wild, but interest in this natural history is growing. Extensive work with lab strains in the last century uncovered the *Saccharomyces* life cycle. When nutrient limited, a diploid yeast cell will form four haploid spores encased in a protective outer layer called the ascus. Confinement within the ascus is thought to enforce mating between products of the same meiotic division, minimizing outcrossing in this stage of the life cycle.

**Methodology/Principal Findings:**

Using a set of *S. cerevisiae* and *S. paradoxus* strains isolated from woodlands in North America, we set up trials in which pairs of asci were placed in contact with one another and allowed to germinate. We observed outcrossing in ∼40% of the trials, and multiple outcrossing events in trials with three asci in contact with each other. When entire populations of densely crowded asci germinated, ∼10–25% of the resulting colonies were outcrossed. There were differences between the species with *S. cerevisiae* having an increased tendency to outcross in mass mating conditions.

**Conclusions/Significance:**

Our results highlight the potential for random mating between spores in natural strains, even in the presence of asci. If this type of mating does occur in nature and it is between close relatives, then a great deal of mating behavior may be undetectable from genome sequences.

## Introduction

**Figure 1:**
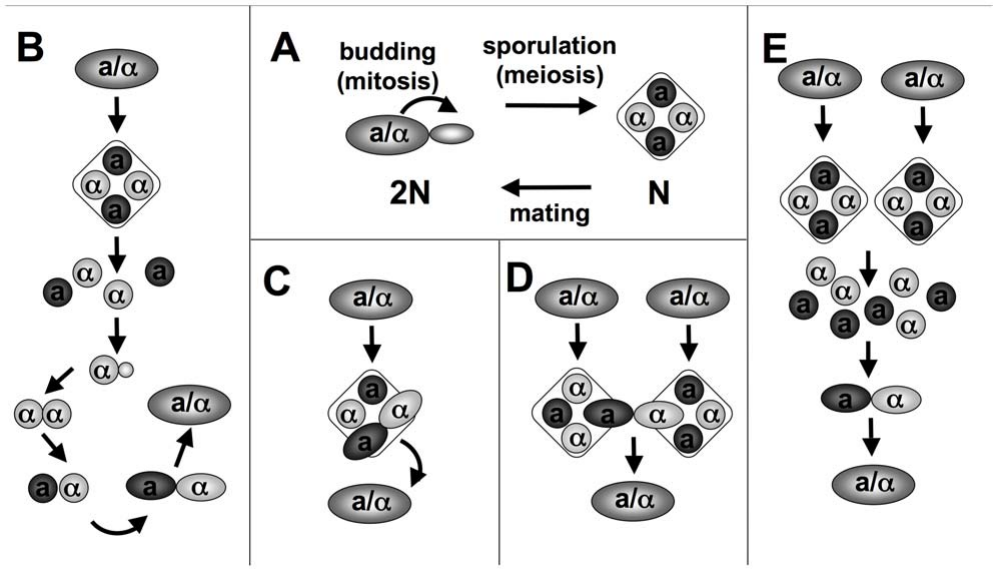
*Saccharomyces* life cycle. **A**) When nutrient limited, diploid cells form four meiotic products, each surrounded by a spore wall, connected via bridges and all encased in one ascus [32]. When conditions permit, spores germinate and bud or mate. Mating occurs between opposite mating types (**a** and **α**) and is either inbreeding (B–C) or outcrossing (D–E). **B**) Mating-type switching: after budding once, a haploid cell can switch its mating type through a highly coordinated gene conversion event [33], then mate with its daughter cell. **C**) Intratetrad mating: spores mate within an ascus. **D**) Intertetrad mating: spores from different asci mate. **E**) Free spores mate with one another at random.

Since the dawn of yeast research, the life cycle (Figure 1) has surprised and fascinated biologists. Early observations of germinating spores mating within an ascus gave yeast the reputation of being a highly inbred organism (for a review of early work, see [1]). That reputation is now supported by population genetic (e.g., [2]), genomic [3], [4], and experimental data [5]. Models of the effects of intra-ascus mating on genetic diversity [6] have lead to suggestions that this form of reproduction purges deleterious mutations while maintaining variation [7]. During meiosis I of spore formation when homologous chromosomes segregate, one copy of the genome becomes associated with each of the mating type alleles (MAT**a** and MAT**α**). Therefore, when spores of the opposite mating type from one ascus mate with each other (forming a MAT**a**/MAT**α** diploid), heterozygosity is maintained only at loci linked to the MAT locus or to a centromere. Genomic observations such as an enrichment of essential genes linked to centromeres seem to support the idea that an advantage of heterozygosity despite intra-ascus mating could be an important force shaping genome organization [5]. However, the frequency of intra-ascus mating in natural populations, and whether outcrossing is even possible in the presence of an ascus, are unknown.

*S. cerevisiae* is found in vineyards [8], as well as alongside *S. paradoxus* in temperate, deciduous woodlands [9]. These two species are strongly genetically post-zygotically isolated [10] and estimated to have diverged over five million years ago [11]. It is thought that these yeasts usually exist in the diploid state [7] and that most mating occurs between germinating spores rather than between vegetative cells [5], [12]. Enzymes produced by flies [13], [14], snails, and mushrooms [15] can digest asci and separate the spores from one another. After a dispersal event, likely via an insect [16]–[18], if compatible partners are present, free spores may mate (Figure 1e) or the diploid state may be restored through mating-type switching (Figure 1b). Without dispersal and ascal digestion, mating should occur in the presence of an ascus (Figures 1c, and as we propose, 1d). While the life cycle allows for various modes of reproduction that can lead to both outcrossing (mating between unrelated spores/cells) and inbreeding (mating between closely related spores/cells), the population genetic structure may still be either recombining or clonal depending on what actually occurs in nature and whether or not there are opportunities for matings among spores from different lineages.

Genomic and population genetic studies of populations of *S. paradoxus* suggest that both inbreeding and outcrossing occur, as signatures of both clonal and recombining population genetic structures have been detected [2], [19]–[21]. Using genomic data from two populations of *S. paradoxus*, Tsai et al. (2008) estimated that when mating occurs, 94% of the time it is intratetrad mating, 5% of the time it is mating type switching, and the remaining 1% of the time it is outcrossing.

Large-scale global studies of *S. cerevisiae* have identified uncultivated genetic lineages, as well as domesticated lineages associated with humans; recombination between these lineages has been detected [21]–[23]. In a comparative genomic study of a clinical, vineyard and lab isolate, recombination was estimated to occur in 1 of every 50,000 cell divisions [3]. However, these studies do not address the frequency of recombination within populations (genetic lineages). There have been two studies of woodland populations of *S. cerevisiae*; in one clonality was observed [24] and in the other, moderate outcrossing was detected in the diploid isolates [25]. In a study of a vineyard population of *S. cerevisiae* in New Zealand, 20% of matings were estimated to be outcrossed [16].

In both species, outcrossing has been detected within populations; however, the stage of the life cycle in which it occurs is unknown. Since direct observations of mating in the wild are not possible, we assayed the frequency of outcrossing in individual trials in which two or three asci were placed in contact with one another, and in mass mating assays of dense sporulated populations using natural strains.

## Materials and Methods

### Yeast Strains

We analyzed six *S. cerevisiae* and six *S. paradoxus* from sympatric woodland populations in eastern North America [24]. Isolates were transformed [26] to create heterothallic strains marked with an antibiotic resistance. Three sets of strains were derived using *ho::KANMX4* [27], *ho::NATMX4*, and *ho::HYGMX4* [28] cassettes. Haploid **a** and **α** strains were isolated by tetrad dissection and subsequently mated to create diploids homozygous for the resistance. Due to the deletion of *HO*, these strains were unable to mating-type switch. In order to determine whether the loss of this ability had an effect on the rate of outcrossing, five of the six original *S. cerevisiae* strains were transformed again, but the antibiotic resistance gene was targeted to an intergenic region near the MAT locus (chr III, 205550–206550). Once transformed, the strains were sporulated and dissected. All spores autodiploidized, confirming the ability to switch mating-type and allowing for the isolation of diploids homozygous for the antibiotic resistance. Table 1 lists the strains used in this study.

**Table 1:** Strain table.

		Heterothallic			Homothallic	
Species	Woodland Strain	Kan^r^	Nat^r^	Hgh^r^	Kan^r^	Hgh^r^
*S. cerevisiae*	YPS681	MZ30	MZ31	MZ57	MZ137	MZ227
	YPS670	MZ32	MZ33	MZ51	MZ373	MZ367
	YPS615	MZ34	MZ35	MZ49		
	YPS133	MZ36	MZ37	MZ55	MZ371	MZ368
	YPS623	MZ38		MZ50	MZ372	MZ369
	YPS630	MZ39		MZ56		MZ370
*S. paradoxus*	YPS664	MZ11	MZ12			
	YPS646	MZ14	MZ13			
	YPS668	MZ73	MZ15			
	YPS642	MZ16	MZ17			
	YPS644	MZ29	MZ28			
	YPS744	MZ18	MZ19			

### Media

Growth occurred in YPD liquid (2% dextrose, 1% yeast extract, 2% peptone) and for the individual trials, sporulation was on solid medium (1% potassium acetate, 0.005% zinc acetate, 2% agar) [29]. Mating assays were performed on SOE plates (1% sucrose, 0.5% dextrose, 0.5% fructose, 0.1% yeast extract, 0.15% peptone, 2% agar) [30]. Media were supplemented with 150 µg/ml G418, 50 µg/ml CloNat, or 400 µg/ml hygromycin, as appropriate.

### Individual Outcrossing Trials

Using a micromanipulator and Zeiss Axioskop FS microscope, two asci from different strains with different antibiotic resistances were placed in pairs in contact with one another on agar plates; 32 pairs (trials) were set up on each plate. Once colonies formed, the mating plates were replica plated to single antibiotic plates to verify the viability of at least one spore in each ascus and then to double antibiotic plates to ascertain outcrossing. Only colonies with viable spores from both asci were included for analysis. At least 25 trials were performed for each pair-wise combination of the twelve heterothallic strains (average 37). All combinations of the five homothallic strains were assayed with at least 32 trials for each (average 56). The frequency of outcrossing for a strain combination was estimated from these multiple trials and considered to be one observation in the statistical analysis. For the three-way outcrossing trials, the same procedure was used, but mating plates were replica plated to three single antibiotic plates, then to three double antibiotic plates. Randomly chosen strain combinations were performed (average 20 trials per combination): fifteen containing 2 *S. cerevisiae* and 1 *S. paradoxus* strains, and 16 containing 1 *S. cerevisiae* and 2 *S. paradoxus* strains.

### Mass Mating

Strains were grown in 3ml of YPD with vigorous shaking for 2–3 days, which led to high rates of sporulation, as measured using a haemocytometer. 300 µl of each of two cultures with different antibiotic resistances were combined, washed, resuspended in 500µl of water, and plated on 60mm SOE plates, which created a dense lawn of asci on the mating plate. Samples were taken to estimate the starting ratio of the strains. *S. paradoxus* strains grew to a slightly lower density; therefore, for interspecies combinations, 350 µl of *S. paradoxus* were combined with 300 µl of *S. cerevisiae*. 24 h later, 3 samples from each mating plate were plated on permissive medium and subsequently replica plated to double antibiotic plates to estimate outcrossing frequency. Mass mating assays were performed once for each of all the pair-wise combinations of the twelve heterothallic strains, as well as for all combinations of the five homothallic *S. cerevisiae* strains.

If outcrossing occurs randomly with a rate of *m*, then 2*mp_a_q_a_* double antibiotic colonies are expected, where *p_a_* and *q_a_* are the frequency of asci of each strain in the combined culture (we assume that all unsporulated cells are diploid and incapable of mating). The starting ratio of the strains, as well as the proportion of asci in a culture, were used to estimate *p_a_* and *q_a_*.

### Statistical Analysis

We performed a two-way ANOVA in JMP with species and assay as the main effects; both were considered fixed. For each strain combination, outcrossing frequency was measured once per assay and treated as one observation in the statistical analysis; therefore strain effects could not be tested. To test whether homothallism had an effect on the frequency of outcrossing, we performed a two-way ANOVA with planned comparisons and considered both mating ability (homo/heterothallism) and assay to be fixed effects.

## Results

**Figure 2:**
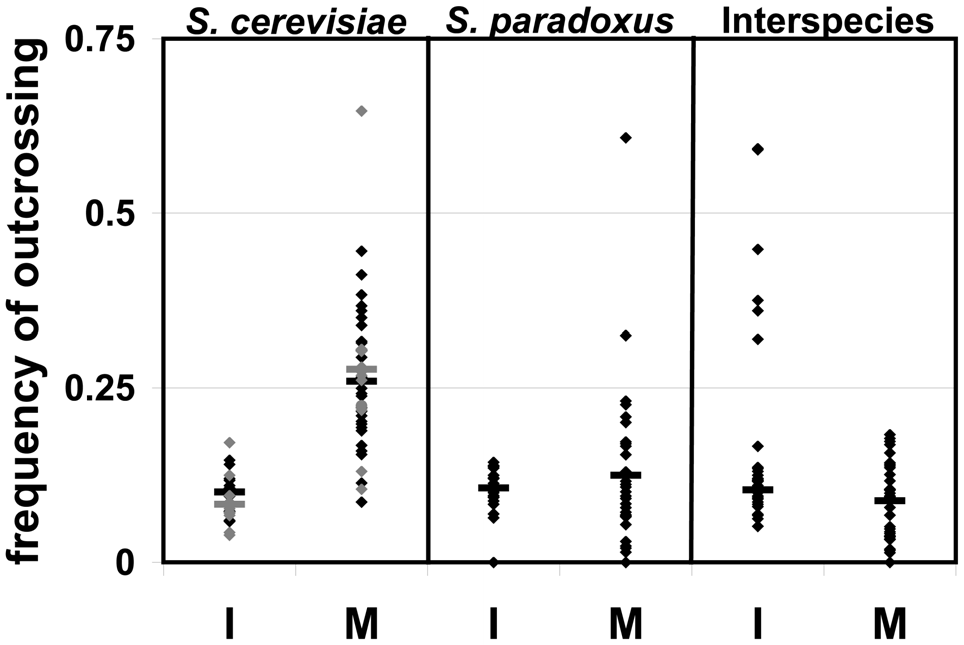
Outcrossing in *Saccharomyces* yeasts. Outcrossing frequency in individual ascus-to-ascus trials (I) and mass mating assays of dense cultures (M); black- assays with heterothallic strains; gray- assays with homothallic strains. Bars represent the overall average; diamonds represent the outcrossing frequency for a given strain combination. For the individual ascus-to-ascus trials, on average, 37 pairs of asci were assayed for each strain combination (one data point on graph); the conservative estimate, which assumes one mating per outcrossed colony, is plotted. In the mass mating assay, for each strain combination, one plate densely covered in asci was allowed to germinate and grow. This plate was sampled and an average of ∼300 resulting colonies were assayed to estimate the outcrossing frequency.

In individual ascus-to-ascus trials, we found high frequencies of outcrossing and no significant differences between intra- and inter-species trials (within *S. cerevisiae*- 40%, within *S. paradoxus*- 43%, interspecies- 42%; see Figure 2). In these trials, since there were 8 spores in total, there were four possible mating events. Our assay could only determine whether or not outcrossing occurred, not the number of times. Therefore, when we observed outcrossing, it meant that at least one and up to four matings were between asci. An observation of 40% of trials exhibiting outcrossing can be interpreted as anywhere from 10–40% of possible matings being between spores from different asci. Since we were unable to determine the actual number of matings, we used the most conservative estimate of each outcrossed colony (only one mating) in our statistical analyses. Using this assumption, the observed rate of outcrossing in the individual trials were *S. cerevisiae*- 10%, *S. paradoxus*- 11%, and interspecies- 10.5%.

**Figure 3:**
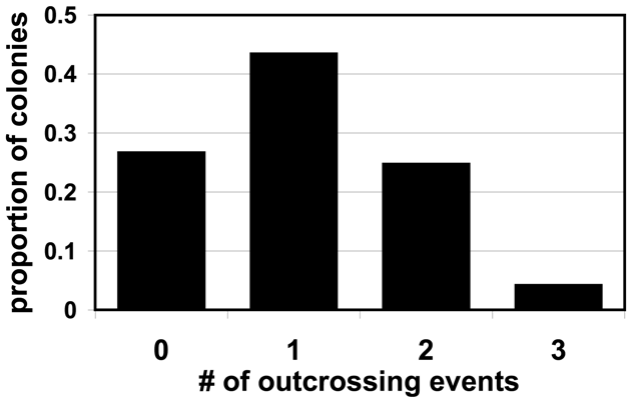
Outcrossing in individual three-way trials. In each trial, three asci, each with a different antibiotic resistance, were placed in contact with one another. After germination and growth, outcrossing was assayed by replica plating to plates with different combinations of antibiotics. In total, 632 trials were conducted. The graph shows the proportion of those trials that contained the number of outcrossing events listed on the x-axis.

Next, we conducted individual trials with trios of asci. In these trials, at least one outcrossing event occurred in 73% of the trials, demonstrating an extremely high rate of outcrossing in yeast when the opportunity is available. We observed one, two and three outcrossing events in 44%, 25% and 4% of the trials, respectively (Figure 3). In these trials, there are 12 spores and 6 possible matings. Using the same logic as above for all three types of observations, this should correspond to 18–50% of possible matings being between spores from different asci.

In entire populations of asci, outcrossing occurred at a relatively high frequency and was significantly more frequent than in individual trials. *S. cerevisiae* outcrossed significantly more than did *S. paradoxus* and interspecies combinations (Sc- 26%, Sp- 13%, interspecies- 9%; see Figure 2). Since we were sampling individuals from an entire population, these values should reflect the percentage of outcrossed matings (or matings between spores from different asci) and can be compared to the conservative estimates from the individual trials.

Overall, we found a significant difference between the outcrossing rates of the species (F = 18.48, *p*<0.0001), significant variation among assay type (F = 22.84, *p*<0.0001) and a significant assay*species interaction (F = 20.28, *p*<0.0001). Using a Tukey's Honestly Significant Difference test, we found that the outcrossing rate of *S. cerevisiae* in mass mating assays was significantly greater than all other assay and species combinations.

To be sure that the high rate of outcrossing we observed was not inflated due to the absence of the ability to switch mating-types, we constructed a second set of *S. cerevisiae* strains that were antibiotic resistant, but still able to switch mating-types, and repeated the assays (Figure 2). We found a decrease in the frequency of outcrossing in the individual ascus-to-ascus trials (homothallic- 33% vs. heterothallic- 40%; with a conservative estimate of matings, homothallic-8% vs. heterothallic-10%) and a slight increase in the mass mating assays of dense populations (homothallic- 27% vs. heterothallic- 26%); however, neither of the differences was significant (planned contrasts, *p* = 0.7774 and *p* = 0.7083, respectively). As in the case with the heterothallic strains, there was more outcrossing in the mass mating assays, (*p* = 0.004). Overall, there were no significant effects due to mating ability (F = 0.2126, *p* = 0.6463) or the interaction between mating ability and assay (F = 0.0011, *p* = 0.9736).

## Discussion

**Figure 4:**
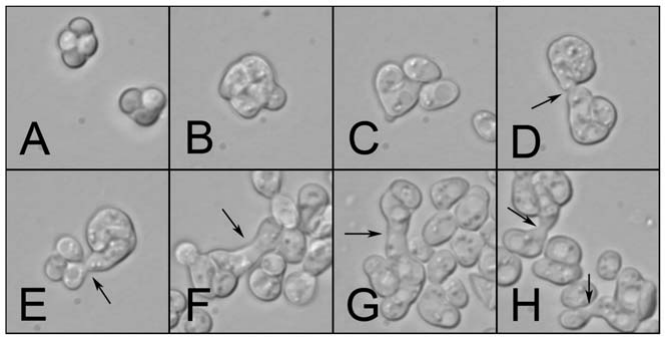
Photos of outcrossing between asci. A–H: Representative photos of sporulated yeast responding to growth medium in a mass-mating assay. Arrows indicate instances of outcrossing. **A**) Sporulated yeast (two asci); **B**) ascus with germinating spores; **C**) four vegetative cells, previously spores from one ascus, two are mating; **D–E**) mating between germinating spores from two different asci; **F–H**) outcrossing and mating in a mass of germinating spores and asci.

Our results clearly demonstrate that the ascus does not physically enforce inbreeding. When environmental conditions become favorable, free yeast spores germinate and then either bud or mate [31]; the same is true when the ascus is still intact (Figure S1; Text S1). Mating may proceed with an ascus mate, or with a germinating spore from a neighboring ascus (Figures 4 and S1). When two asci are placed in contact with each other, at least one outcrossing event occurs ∼10–40% of the time, and when three asci are placed in contact, at least one outcrossing event occurs ∼20–50% of the time. When an entire dense population of sporulated yeast germinate, outcrossing occurs ∼10–25% of the time. The higher rate of outcrossing in mass mating may be due to the density of spores and/or the difference in the availability of nutrients. Since so little is known about the ecology of *Saccharomyces* yeasts, it is uncertain which of the two assays more closely mimics natural conditions. Because intact asci probably don't migrate, and therefore neighboring asci are likely to be closely related (potentially descendants from the same mother cell), it is unclear whether this form of mating would impact the frequency of random mating in a population and therefore have an effect on the population genetic structure. This may be a reason why inter-ascus mating has been overlooked in other studies investigating the frequency of intratetrad mating [4], [5].

Tsai et al. used genomic data from 8 and 12 strains from a Far Eastern and a European population of *Saccharomyces paradoxus*, respectively, to estimate the frequency of asexual and sexual reproduction by comparing the genetic diversity presumably contributed by mutation and recombination. They used their estimates of *rho* to calculate the frequency of each type of sexual event: mating-type switching, intra-ascus mating, and random mating. By comparing diversity surrounding the *MAT* locus to the rest of chromosome III, they estimated the frequency of intra-ascus mating (at all loci other than *MAT*, each form of mating has a different effect on heterozygosity). Once they estimated the frequency of intra-ascus mating to be 94%, they added 1% for outcrossing/random mating (using a previous estimate based on observed heterozygosity in a population survey) and presumed the last 5% of the time mating-type switching occurred. They did not consider the possibility of inter-ascus mating. If two asci were closely related (descendants from the same mother cell), inter-ascus mating would be another form of inbreeding that could have contributed to the decrease in diversity on chromosome III, and would not be differentiated from intra-ascus mating in their analysis. Conversely, if two asci were unrelated and inter-ascus mating occurred, then it would be counted as part of the estimate of random mating (outcrossing).

In another study that investigated intra-ascus mating, Taxis et al. conducted a series of experiments to show that based on nutrient availability the number of spores produced in an ascus is a self-organizing system and may be optimized so that yeast can return to the diploid state without intervening mitotic divisions. One experiment in their study is particulary relevant to the discussion here. Since all centromeres are in effect linked to the *MAT* locus, when intra-ascus mating occurs, heterozygosity is also preserved at sequences closely linked to centromeres (see Figure 7A in their paper). To assay intra-ascus mating, Taxis et al. constructed a yeast strain that was heterozygous (hph^r^/kan^r^) at a locus near a centromere, and heterozygous (+/nat^r^) at a locus for an essential mitosis gene on a different chromosome. A culture of asci was exposed to growth medium and subsequently sampled to determine the antibiotic resistances of the sample colonies. The authors found that 75%–80% of the sampled colonies contained all three markers, which they interpreted as resulting from intra-ascus mating between non-sister spores; the remaining 20–25% of the diploid colonies did not contain all three antibiotic markers. Some of these remaining colonies had clonat resistance (which by definition means they were heterozygous because nat^r^/nat^r^ could not divide mitotically) and had only one of the other markers (hph^r^/hph^r^ or kan^r^/kan^r^). These colonies were interpreted as being the result of “mating upon germination but not between sister spores”. They also observed colonies that lacked clonat resistance (+/+) and had any combination of the other two markers. They interpreted these colonies as deriving from “other types of mating”. All of the matings they observed that did not have all three markers are consistent with inter-ascus mating (although colonies that had only kan or hph resistance are consistent with mating-type switching as well). If one lists all the different mating combinations possible in the previously described experiment (two loci on different chromosomes, with two alleles per locus), there are 16 possibilities, four of which cannot grow and survive because they lack an essential mitotic gene. Another four of the sixteen should contain all three antibiotic markers. The final eight constitute the combinations lumped into the two “other” categories in their analysis. If inter-ascus mating did occur in their experiment, and when it did, it occurred randomly with respect to their antibiotic markers, then the 20–25% that they observed that did not contain all three markers could potentially represent half of the inter-ascus mating events. If that were true, then the estimate of inter-ascus mating would be similar to the frequency we observed in our experiments (up to 40%).

Although some studies of yeast populations have found evidence for outcrossing and recombination, others have not. We see three ways to reconcile our observation of frequent mating between spores from different asci with the apparent rarity of outcrossing among the genome sequences of natural isolates. The first is that the estimates based on comparative genomics may either underestimate the frequency of inter-ascus mating (as explained above) or may not be relevant to within population dynamics. The estimate of 1 recombination event in 50,000 cell divisions provided by Ruderfer et al. is based on comparing strains from different global and ecological lineages (clinical, vineyard, lab) and may accurately reflect inter-lineage dynamics; however, it may not reflect outcrossing and mating dynamics within lineages and populations in vineyards and woodlands. The second possibility is that although the ascus structure does not prevent matings between asci, due to ecological circumstances in some populations, ascus density is low enough that they are rarely in contact. The final possibility is that, because of intervening clonal growth between sexual events, neighboring asci are usually closely related, so mating between them has little or no detectable effect on population genetic structure. While much of the population and genomic data have supported yeast's reputation as an inbred organism, the ascus should no longer be considered the chief cause.

## Supporting Information

Figure S1 Photos of the time course in a mass-mating assay with *S. cerevisiae*. A) 0 hours: intact asci that are being placed in permissive medium. B) 3 hours later, the asci appear flat and the spores begin to swell. C) 4 hours after being placed into permissive medium, the spores are germinating out of the ascus. D) 5 hours after initial exposure, budding cells are visible, as are masses of cells that were once asci. E–H) In the center of each photo are examples of outcrossing between two asci, all were taken at 5 hours.
(0.27 MB TIF)

Text S1 (0.05 MB DOC)

## References

[1] Guilliermond A, Tanner FW (1920). The Yeasts.

[2] Johnson L, Koufopanou V, Goddard M, Hetherington R, Schäfer S (2004). Population genetics of the wild yeast *Saccharomyces paradoxus*.. Genetics.

[3] Ruderfer DM, Pratt SC, Seidel HS, Kruglyak L (2006). Population genomic analysis of outcrossing and recombination in yeast.. Nature Genetics.

[4] Tsai IJ, Bensasson D, Burt A, Koufopanou V (2008). Population genomics of the wild yeast *Saccharomyces paradoxus*: Quantifying the life cycle.. Proceedings of the National Academy of Sciences of the USA.

[5] Taxis C, Keller P, Kavagiou Z, Jensen LJ, Colombelli J (2005). Spore number control and breeding in *Saccharomyces cerevisiae*: a key role for a self-organizing system.. Journal of Cell Biology.

[6] Zakharov IA (2005). Intratetrad mating and its genetic and evolutionary consequences.. Genetika.

[7] Knop M (2006). Evolution of the *hemiascomycete* yeasts: on life styles and the importance of inbreeding.. BioEssays.

[8] Mortimer R, Polsinelli M (1999). On the origins of wine yeast.. Research in Microbiology.

[9] Naumov GI, Naumova ES, Sniegowski P (1998). *Saccharomyces paradoxus* and *Saccharomyces cerevisiae* are associated with exudates of North American oaks.. Can J Microbiol.

[10] Liti G, Barton DB, Louis EJ (2006). Sequence diversity, reproductive isolation and species concepts in Saccharomyces.. Genetics.

[11] Kellis M, Patterson N, Endrizzi M, Birren B, Lander E (2003). Sequencing and comparison of yeast species to identify genes and regulatory elements.. Nature.

[12] Maclean CJ, Greig D (2008). Prezygotic reproductive isolation between *Saccharomyces cerevisiae* and *Saccharomyces paradoxus*.. BMC Evolutionary Biology.

[13] Reuter M, Bell G, Greig D (2007). Increased outbreeding in yeast in response to dispersal by an insect vector.. Current Biology.

[14] Coluccio AE, Rodriguez RK, Kernan MJ, Neiman AM (2008). The yeast spore wall enables spores to survive passage through the digestive tract of *Drosophila*.. PLoS One.

[15] Bevan EA, Costello WP (1964). The preparation and use of an enzyme which breaks open yeast asci.. Microbial Genetics Bulletin.

[16] Goddard MR, Anfang N, Tang R, Gardner RC, Jun C (2009). A distinct population of *Saccharomyces cerevisiae* in New Zealand: evidence for local dispersal by insects and human-aided global dispersal in oak barrels.. Environmental Microbiology.

[17] Lachance MA, Gilbert DG, Starmer WT (1994). Yeast communities associated with *Drosophila* species and related flies in eastern oak-pine forests: a comparison with western communities.. Jounal of Industrial Microbiology.

[18] Phaff HJ, Knapp EP (1956). The taxonomy of yeasts found in exudates of certain tress and other natural breeding sites of some species of *Drosophila*.. Antonoie van Leeuwenhoek.

[19] Koufopanou V, Hughes J, Bell G, Burt A (2006). The spatial scale of genetic differentiation in a model organism: the wild yeast Saccharomyces paradoxus.. Philisophical Transactions of the Royal Society B.

[20] Kuehne HA, Murphy HA, Francis CA, Sniegowski PD (2007). Allopatric divergence, secondary contact, and genetic isolation in wild yeast populations.. Current Biology.

[21] Liti G, Carter DM, Moses AM, Warringer J, Parts L (2009). Population genomics of domestic and wild yeasts.. Nature.

[22] Aa E, Townsend JP, Adams RI, Nielsen KM, Taylor JW (2006). Population structure and gene evolution in Saccharomyces cerevisae.. FEMS Yeast Research.

[23] Schacherer J, Shapiro JA, Ruderfer DM, Kruglyak L (2009). Comprehensive polymorphism survey elucidates population sturcture of *Saccharomyces cerevisiae*.. Nature.

[24] Kuehne HA *Genetic Population Structure and Biogeography of Natural Saccharomyces Populations*, in *Department of Biology*..

[25] Katz Ezov T, Boger-Nadjar E, Frenkel Z, Katsperovski I, Kemeny S (2006). Molecular-genetic biodiversity in a natural population of the yeast *Saccharomyces cerevisiae* from “Evolution Canyon”: Microsatellite polymorphism, ploidy and controversial sex status.. Genetics.

[26] Gietz R, Woods R (2002). Transformation of yeast by the Liac/Ss Carrier Dna/Peg method.. Methods in Enzymology.

[27] Wach A, Brachat A, Pohlmann R, Philippsen P (1994). New heterologous modules for classical or PCR-based gene disruptions in *Saccharomyces cerevisiae*.. Yeast.

[28] Goldstein A, McCusker J (1999). Three new dominant drug resistance cassettes for gene disruption in *Saccharomyces cerevisiae*.. Yeast.

[29] Rose MD, Winston F, Hieter P (1990). Methods in Yeast Genetics: A Laboratory Course Manual.

[30] Murphy HA, Kuehne HA, Francis CA, Sniegowski PD (2006). Mate choice assays and mating propensity differences in natural yeast populations.. Biology Letters.

[31] Joseph-Strauss D, Zenvirth D, Simchen G, Barkai N (2007). Spore germination in *Saccharomyces cerevisiae*: global gene expression patterns and cell cycle landmarks.. Genome Biology.

[32] Coluccio A, Neiman A (2004). Interspore bridges: a new feature of the Saccharomyces cerevisiae spore wall.. Microbiology.

[33] Haber J (1998). Mating-type gene switching in Saccharomyces cerevisiae.. Annual Review of Genetics.

