# Supplementary figures and images for: Yeast Sex: Surprisingly High Rates of Outcrossing between Asci
Source: PLoS One. 2010 May 5;5(5):e10461. doi: 10.1371/journal.pone.0010461 (PMC2864747; doi:10.1371/journal.pone.0010461)

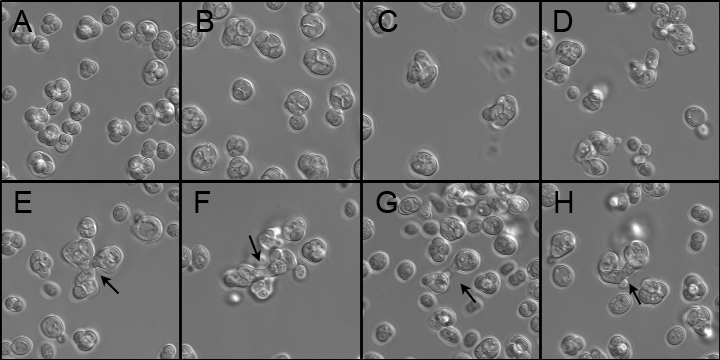

Supplement: Figure S1 — Photos of the time course in a mass-mating assay with S. cerevisiae. A) 0 hours: intact asci that are being placed in permissive medium. B) 3 hours later, the asci appear flat and the spores begin to swell. C) 4 hours after being placed into permissive medium, the spores are germinating out of the ascus. D) 5 hours after initial exposure, budding cells are visible, as are masses of cells that were once asci. E–H) In the center of each photo are examples of outcrossing between two asci, all were taken at 5 hours. (0.27 MB TIF) [file pone.0010461.s001.tif]
