# Supplementary material for: Yeast Sex: Surprisingly High Rates of Outcrossing between Asci
Source: PLoS One. 2010 May 5;5(5):e10461. doi: 10.1371/journal.pone.0010461 (PMC2864747; doi:10.1371/journal.pone.0010461)
Supplement: Text S1 — (0.05 MB DOC) [file pone.0010461.s002.doc]

We were not the first to observe what happens when sporulated yeast germinate. The following is an exerpt from “The Yeasts” by Alexandre Guilliermond and Fred Tanner (1920):

“Let us start this discussion with *Saccharomyces cerevisiae*… In the first phases of germination, the ascospores undergo a swelling, but the wall subsists. This swelling is so great that the ascospores, by means of pressure which they exert on one another, give the impression that the asc[us] is chambered. In fact, the walls of the ascospores enter into intimate contact, and often they fuse completely in such a way that there are really walls in the asc[us] which then becomes a cell with many chambers. During this time the wall of the asc[us] becomes thinner and finally breaks. It acts as a plaited veil which retains the ascospores, or is completely absorbed by the ascospores. Each ascospore then takes the form of an ordinary vegetative cell.” pp29-30
